# Call for a Dedicated European Legal Framework for Bacteriophage Therapy

**DOI:** 10.1007/s00005-014-0269-y

**Published:** 2014-02-06

**Authors:** Gilbert Verbeken, Jean-Paul Pirnay, Rob Lavigne, Serge Jennes, Daniel De Vos, Minne Casteels, Isabelle Huys

**Affiliations:** 1 Laboratory for Molecular and Cellular Technology, Burn Wound Centre, Queen Astrid Military Hospital, Brussels, Belgium; 2 Faculty of Pharmaceutical and Pharmacological Sciences, KU Leuven, Leuven, Belgium; 3 Laboratory of Gene Technology, KU Leuven, Leuven, Belgium; 4 Burn Wound Centre, Queen Astrid Military Hospital, Brussels, Belgium; 5 Centre for Intellectual Property Rights, KU Leuven, Leuven, Belgium

**Keywords:** Bacteriophage, Therapy, Human, European, Regulatory, Legal, Legislation

## Abstract

The worldwide emergence of antibiotic resistances and the drying up of the antibiotic pipeline have spurred a search for alternative or complementary antibacterial therapies. Bacteriophages are bacterial viruses that have been used for almost a century to combat bacterial infections, particularly in Poland and the former Soviet Union. The antibiotic crisis has triggered a renewed clinical and agricultural interest in bacteriophages. This, combined with new scientific insights, has pushed bacteriophages to the forefront of the search for new approaches to fighting bacterial infections. But before bacteriophage therapy can be introduced into clinical practice in the European Union, several challenges must be overcome. One of these is the conceptualization and classification of bacteriophage therapy itself and the extent to which it constitutes a human medicinal product regulated under the European Human Code for Medicines (Directive 2001/83/EC). Can therapeutic products containing natural bacteriophages be categorized under the current European regulatory framework, or should this framework be adapted? Various actors in the field have discussed the need for an adapted (or entirely new) regulatory framework for the reintroduction of bacteriophage therapy in Europe. This led to the identification of several characteristics specific to natural bacteriophages that should be taken into consideration by regulators when evaluating bacteriophage therapy. One important consideration is whether bacteriophage therapy development occurs on an industrial scale or a hospital-based, patient-specific scale. More suitable regulatory standards may create opportunities to improve insights into this promising therapeutic approach. In light of this, we argue for the creation of a new, dedicated European regulatory framework for bacteriophage therapy.

## Introduction

Antimicrobial resistance is a key twenty-first century global health challenge (Cooper and Shlaes 2011; Kutateladze and Adamia 2010). The potential of bacteriophages for treating (multi-drug resistant) bacterial infections has been acknowledged for decennia (Brüssow 2005; Gill and Hyman 2010; Górski et al. 2009a, b; Maura and Debarbieux 2011; Pirnay et al. 2012) and bacteriophage research is being performed intensively worldwide (Ackermann 2012). Bacteriophage therapy was developed mainly in Eastern Europe (Poland) and the former Soviet Republics (Georgia and Russia). A handful of clinical trials have been performed in those countries, as well as in the United States and India (Bruttin and Brüssow 2005; Monk et al. 2010); however, most of these studies were not carried out according to modern, evidence-based standards of medical research (Parracho et al. 2012). Today, a small number of clinical trials have been carried out and/or are ongoing and bacteriophage therapy is being applied in clinical settings under the purview of specific national regulatory frameworks and/or the Helsinki Declaration (Górski et al. 2009a, b; Kutter et al. 2010).

The lack of a smooth (re-) introduction of bacteriophage therapy in Europe is related to several obstacles within the current European Regulatory Framework (Brüssow 2012; Pirnay et al. 2011; Verbeken et al. 2012; Wright et al. 2009). Meanwhile, the UK’s Medicines and Healthcare Products Regulatory Agency has approved a bacteriophage clinical trial (Pirnay et al. 2011), which is now ongoing. In this context, bacteriophages used as therapeutics are considered “biological medicinal products” by European regulators. In the United States, such bacteriophage-based products are handled by the FDA division for vaccines and related product applications (Parracho et al. 2012). This suggests that a non-specific, technical and stringent legislative pharmaceutical framework is likely to be introduced into the field of natural bacteriophage therapy in the near future. Hospitals using bacteriophage-based products to treat hospitalized patients—many of which hospitals have used these products for many years—must now meet the stringent requirements pertaining to “true” human medicinal product development. This is likely to be destructive for the non-profit (tailored) hospital-based use of therapeutic bacteriophages as well as for small and medium enterprises lacking the necessary financial resources to fund the full product development cycle for bacteriophage-based products (Pirnay et al. 2012; Thiel 2004).

Currently, the regulatory aspect of bacteriophage therapy is understudied. No technical, scientific arguments currently exist addressing the question of whether and to what extent bacteriophages fit within the actual definitions and procedures of the existing regulatory framework for human medicinal products in Europe.

This study investigates the scientific arguments related to the classification of bacteriophages as human medicinal products under the current European regulatory framework. The core of the discussions was the European legislation relevant to the therapeutic (*anti*-*bacterial*) use of natural (*not genetically modified*) bacteriophages in humans.

The aim of the study was to evaluate whether the current European regulatory framework for human medicinal products needs to be adapted with regard to an eventual (re-) introduction of bacteriophage therapy into the European Union.

## Methodology

The research focuses on the application of *natural* bacteriophages in a *therapeutic* context. Other possible fields of applications for bacteriophages (e.g., prevention of infections; use as vaccines; use as diagnostic tools; use as a tool to influence cancer or to decontaminate skin grafts) were excluded.

To investigate the extent to which the concept of bacteriophage therapy does or does not fit into the current European regulatory framework for human medicinal products, the existing biomedical-economic literature was reviewed and in-depth interviews with 35 key informants with knowledge and/or regulatory expertise of bacteriophages were carried out. Participants were selected using purposive sampling. The experts represent different stakeholder groups, including industry (11), academia (18), hospitals (5) and competent authorities (1). The interviewees were based in Belgium (15), France (8), United States (3), United Kingdom (2), Georgia (2), Germany (1), Poland (1), Portugal (1), Switzerland (1) and The Netherlands (1).

**Fig. 1:**
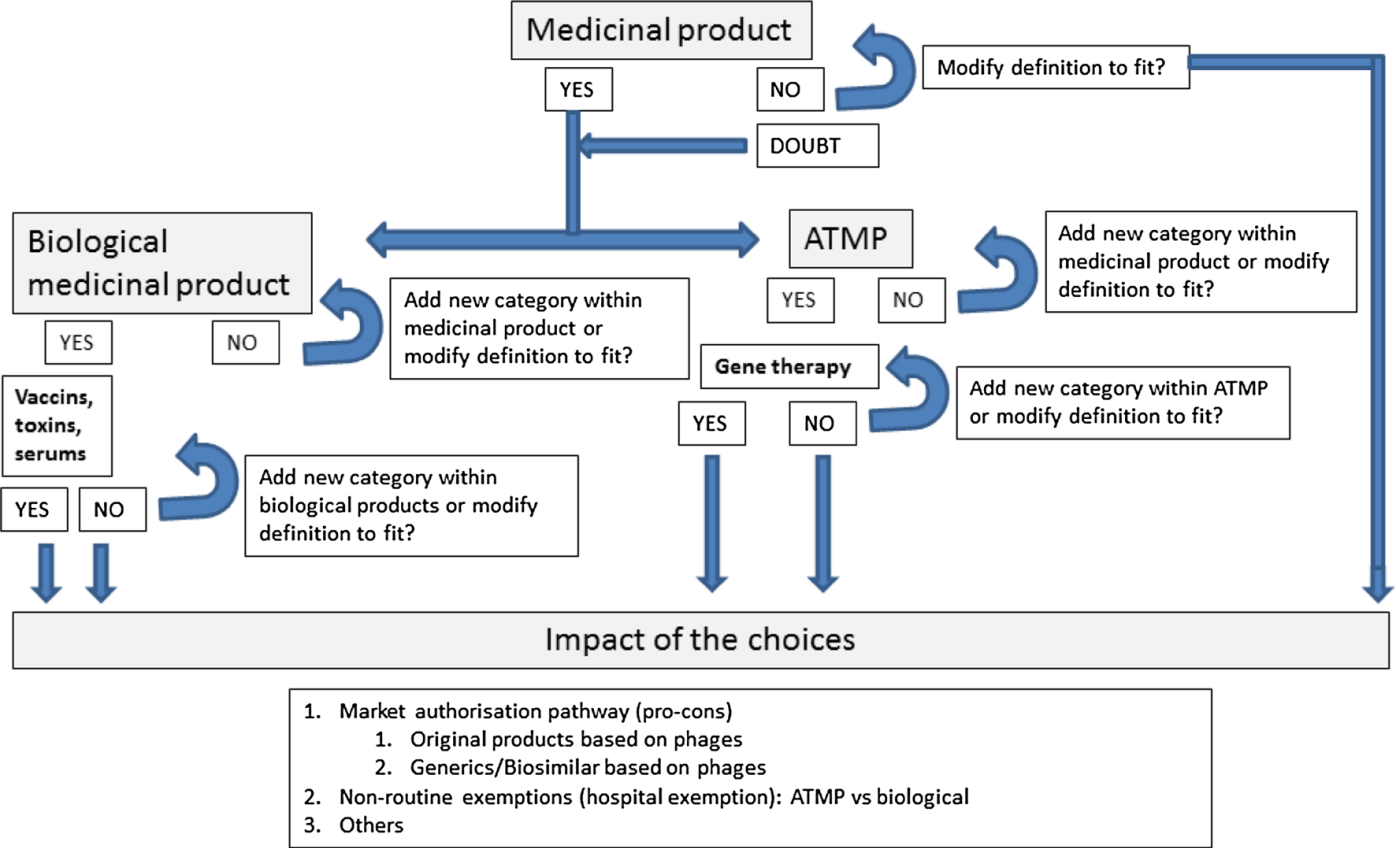
Overview of methodological approach of the interviews

The interview was based on a standardized questionnaire. Three definitions from the existing European regulatory framework were presented to the interviewees (Boxes 1–3): the general definition of a medicinal product, the definition of a biological medicinal product, and the definition of an Advanced Therapy Medicinal Product (ATMP). The interviewees were asked to comment on how bacteriophages did or did not fit into the wordings of the presented definitions (see Fig. 1). Beside these three main definitions, the following topics where also discussed: the definition of a bacteriophage, whether (or not) a bacteriophage is in fact a living entity, therapeutic quality and safety issues, application methodologies, possible side effects of bacteriophages, differences/similarities of bacteriophage therapy versus antibiotic therapy, marketing authorization pathways, the hospital exemption issue and intellectual property aspects. The interviewees were asked to formulate conclusions about whether (or not) the current European regulatory framework is sufficient, needs to be adapted or whether there is a need for a new, dedicated framework specifically for bacteriophages.

The interviews were qualitatively analyzed and consistent themes and patterns were identified. Due to the complexity of the interview data, results were processed and analyzed using non-computational qualitative methodology (Silverman 2010).

## Results

This chapter summarizes the answers/the reflections of the interviewees in relation to the questions asked. These answers, reflections or statements do not necessary reflect the position of the authors of this paper. Literature-references are not included in this chapter since it is not known to the authors from what (publication) background the interviewees were answering the questions.

### Bacteriophages as Human Medicinal Products under the European Regulatory Framework

#### Arguments Related to Bacteriophages and the Definition of a Human Medicinal Product (see Box 1)

The definition of a human medicinal product (Art. 1 of Directive 2001/83/EC) refers to a “substance” (or a combination of substances) presented with particular therapeutic properties and used in therapeutic contexts. Such a substance is also defined in the Directive (Box 1) and is perceived to be “any matter irrespective of origin”, with some additional examples. The definition of this referred substance is so broadly defined that it includes natural bacteriophages used as antimicrobial agents within human beings. The definition could even be taken to cover a physician, since a physician is also “presented to patients as having properties for treating disease”.

In view of the particular examples of substances (Box 1), opinions differ on what a natural bacteriophage really is. A bacteriophage can be considered a microorganism—or not—and as living—or not. Differences at this conceptual level are important when considering a potential classification of natural bacteriophages in the existing legal framework for human medicinal products.

In the case that a bacteriophage is considered a microorganism, we can refer to it as a bacterial virus, a microbe or some other organism. According to classical taxonomical terminology, a bacteriophage is indeed a virus. A virus outside a bacterium is called a “virion” (an “intermediate phase”). “Virions” can be compared to spores or sperm cells. A spore is not a plant, and a sperm cell is not a human being. Once the virion is inside the bacterium, this bacterium is no longer the same cell. The virion takes over the essential elements and processes within the bacterium. The changed (infected) cell could thus be called a “viral cell”. It is the virus-cell combination that then produces the bacteriophages (virions). In this view, the bacteriophage together with its bacterium can be considered to be *one* microorganism, since a bacteriophage has no existence without that bacterium. The *bacteriophage*-*bacterium combination* could be classified as a new taxonomic entity.

On the other hand, there are arguments supporting the idea that a bacteriophage is not a microorganism since a bacteriophage has no “organs” and requires a cell machinery to be “alive”. According to this logic, a bacteriophage can be considered as *derived from* a microorganism.

With respect to its replicating nature, a bacteriophage is perceived as a biological entity that, by interacting with its (biological) environment, is capable of replicating and evolving as an independent, self-replicative particle. But others do not consider a bacteriophage self-replicative since a bacteriophage needs a host (a biological system) to self-replicate.

##### Is a Bacteriophage Living?

Some do not consider a bacteriophage as a living entity since it lacks the most basic component of a biological system, namely, a cell (the biologic basic entity). A cell is an open thermo-dynamic system with a constant material and energy flow. Being alive implies a status with a “anti-entropic effect”. This contrasts with a bacteriophage as a (classically defined) virus consisting only of (static) proteins and nucleic acids. Others consider bacteriophages as living entities. Due to it being a very small entity—a capsuled single-stranded DNA—(the entire DNA of) a bacteriophage was one of the first molecules to be synthesized in the laboratory. Although very simple in design, this piece of DNA is not functional when introduced as such into a bacterium. Therefore, arguments can be found to qualify bacteriophages as “living”. Viruses are, after all, part of the “tree of life”.

##### Therapeutic Action of Bacteriophages

Bacteriophages can “treat or prevent” a disease in human beings and they “restore, correct or modify physiological functions by exerting pharmacological, immunological or metabolic actions” as described in the Directive (Box 1). Different aspects can be considered with respect to the exact mode of action of a therapeutic bacteriophage. Bacteriophages can *restore* physiological function and the original endemic flora by controlling the pathogens present there. In this way, they can restore balance to out-of-control systems. In cases where different bacteria are involved, bacteriophages can generate a competitive exclusion by specifically attacking a particular bacterium, thus rebalancing the ecosystem.

Another mode of action of bacteriophages relates to their capacity to effectively *modify* human physiological functions, be it in an indirect way, by destroying the bacteria. In this sense, they are comparable with antibiotics. *Immunological* actions can be attributed to bacteriophages by specifically boosting the human immune system. Even a *metabolic action* of bacteriophages can be observed, since bacteriophages interact with the microbial parts of the human body, correcting or modifying physiological functions by killing off pathogenic microorganisms. In addition, bacteriophages take over the bacterial metabolism. Bacteriophages can also generate a *pharmacological* action since bacteriophages are not only antimicrobials but can also suppress inflammation caused by infection.

Finally, bacteriophages can be used as a medical diagnostic tool (Box 1), as was the case when they were used in *salmonella* testing during *salmonella* outbreaks and fast plaque testing for tuberculosis.

##### Route of Administration of a Bacteriophage-Based Product

There is a lack of scientific evidence about the most optimal application format and methodology for bacteriophage therapy. The external (topical) or oral use of bacteriophages should pose no problems. The preferable application method, however, is intra-peritoneal or intra-muscular. Bacteriophages are then released into the bloodstream very slowly, gradually and at low levels. In this way, the immune system is stimulated much less than it would be were the bacteriophages to be directly injected intravenously. Once the bacteriophages are at the point of action, they will auto-amplify as needed. Bacteriophages have widely been used intravenously. For instance, the intravenous anti-staphylococcal bacteriophages produced at the Eliava Bacteriophage Institute’s industrial department have been used across the whole Soviet Union from the end of 1970s through the end of 1980s for treatment of septic infections in humans (children and adults) caused by multiple drug-resistant *Staphylococcus aureus*. However, there is some sense in not administering bacteriophages intravenously, particularly because bacteriophages are likely to be filtered out by the immune system almost immediately using this method. In an effort to prevent this, one could try to cover the bacteriophages with molecules, making them invisible to the immune system. However, after this manipulation, the bacteriophages can no longer be considered “natural” bacteriophages. Ultimately, while promising as an avenue for further research, bacteriophages may not be suited to treating kidney or liver infections since maintaining adequate bacteriophage concentrations to treat at these locations is probably infeasible.

##### Possible Side Effects of Bacteriophage Therapy

Predicted side effects are very few and mostly depend on the time of administering the bacteriophages, the applied amount of bacteriophages, the type of bacteriophages used, the format of application, and whether the bacteriophages are administered as cocktails.

With respect to genetic (carcinogenic) consequences related to bacteriophage therapy, gene transfer cannot be totally excluded, but will probably only happen at a very low frequency.

Bacteriophages can cross the blood–brain barrier, but no known specific side effects related to this have been reported.

Immunological response at the moment of treatment and immunization against the bacteriophages when used in the long run could also be possible. This phenomenon is not likely to appear when the treatment period is (very) short. This is why repetitive treatment at intervals of several weeks or months (with the same bacteriophages) should be avoided. When using bacteriophage cocktails in a particular therapy, bacteriophages must be changed or updated frequently and broad-spectrum cocktails must be composed of the least possible number of bacteriophages.

The use of therapeutic bacteriophages will lead to a quick and in some cases quit massive destruction of the bacterial cells involved. At worst, this massive and total lysis of bacteria and the subsequent release of toxins could generate potentially life-threatening reactions such as endotoxin shock, mechanical osmotic effects or respiratory symptoms. The use of small quantities of bacteriophages at once is necessary in order to avoid the large-scale release of toxins. In addition, a first, limited amount of bacteriophages prior to a higher therapeutically relevant dose can prevent large-scale toxin release because the initial bacteriophages destroy the bacteria before they multiply massively.

A side effect of bacteriophage cocktails in particular is the risk of recombination that can occur within a bacteriophage, ending all control over the process. Recombining the genetic information within bacteriophages can modify the original bacteriophages. This happens in nature and is being studied in labs; however, more modeling studies are necessary to fully explore this.

It is clear that the long-term consequences of bacteriophage therapy remain partly unknown, especially in view of the resistance development. Although bacteriophages can adapt and evolve along bacterial changes, research related to the development of resistance in general and research on bacteriophages more specifically is therefore necessary. We must treat carefully and draw on lessons learned in the past from the development cycle of antibiotics, which progressed without any profound, thorough risk assessment. For bacteriophages, the (environmental) risk assessment for (non-human) medical use is also important due to problems that may arise from the massive use of bacteriophages in, e.g., the veterinary, bio-agricultural industry, as was and continues to be the case for antibiotics use in that industry.

#### Views Related to Bacteriophages and the Definition of a Biological Medicinal Product (see Box 2)

The definition of a Biological Medicinal Product refers to the active substance as a biological substance produced or extracted from a particular biological source. Active products used in natural bacteriophage therapy can be classified under the definition of a Biological Medicinal Product.

This is the case for several reasons. First, a natural bacteriophage itself can be perceived as a *biological* *substance*. Such bacteriophages can be produced by or extracted from a biological *source*, as proclaimed in the Directive. The bacterium itself can be seen as the biological source. Other possible biological sources are the initial ecological combination ‘bacteriophage-bacterial host’, or the bacteriophage itself, which enters a bacterial cell, interacts with it and replicates. Even the wound fluid of the patient or the wastewater out of which bacteriophages can be extracted could be viewed as possible biological sources.

It is also possible to view a bacteriophage as not extracted from but *made by* the bacterial cell. The bacteriophage lyses the bacterial cell and releases itself from its host. A bacteriophage has a self-replicating nature, but it can only reproduce (or make) itself when present in a bacterium, namely, a very bacterial-specific host or the biological system to which it belongs.

One could also consider the endozymes produced by the bacteriophage as active biological *substances*. Such endozymes cause lysis of the bacterium and originate from the bacteriophages as a biological *source*.

When bacteriophages are considered as human medicinal products, the *starting material* (as indicated in Directive 2001/83/EC) for producing the therapeutic bacteriophage product must be a substance of biological origin (Box 2). The exact meaning of that starting material can differ. One can consider a microorganism as the starting material for producing a therapeutic bacteriophage, or a particular substance, produced by a microorganism. Another view identifies two types of starting materials for producing therapeutic bacteriophages, namely, “virions” and bacteria, forming bacterio-viruses. Yet another approach is simply to characterize a bacteriophage’s parent as its substance of origin.

##### Physico-Chemical-Biological Testing of Bacteriophages

With respect to the characterization and determination of the *quality* of a bacteriophage-based product (Box 2), it could be argued that a combination of physico-chemical-biological testing is required, together with testing of the production process and its control, as described in the Directive (Box 2). However, the exact meaning of physico-chemical-biological testing in view of bacteriophage therapy needs to be clarified, particularly in relation to the required documentation package for bacteriophage therapy.

To generate a qualitative effect of therapeutic bacteriophages, the first requirement is to assess the underlying therapeutic problem of the patient, namely to identify the problematic bacterial strain so that the right corresponding (most effective) therapeutic bacteriophage can be selected.

Once selected, the bacteriophage-bacterium interaction (the *efficacy*) needs to be evaluated in vitro. Electron microscopy can be helpful in documenting the interaction bacteriophage-bacterium.

Bacteriophages need to be characterized in view of the specific *morphotype*. Maximal *molecular characterization* of the bacteriophage genome is mandatory to confirm the absence of known toxic genes or to confirm the absence of known antibiotic-resistant genes, but not for each batch produced (only for the master stock). In view of the genetic testing of bacteriophages, it could be useful to have a microchip formulation that could be used to test any cocktail to ensure that it is not carrying a pathogenicity island. Full genetic sequencing can, however, lead to false safety statements since, even when a bacteriophage is fully sequenced, half of its genome (and/or related functions) remains unknown. The presence of *lysogenic bacteriophages* must be maximally excluded. In any case, it is also important to point out that a bacteriophage that lacks any lysogenic component can acquire one from a lysogenic bacteriophage that is already present in the body. Bacteriophages arising from host bacteria with the lowest level of emerging *mutations* must be chosen for the production of bacteriophage preparations. When possible, bacteriophages should be produced in a *non*-*pathogenic bacterial host*, and that host must be sequenced as well. This issue is less (or not) relevant when bacteriophages are grown on the patient’s own bacteria. In order to avoid genetic alterations, it would be wise *not* to *scale up* the production of bacteriophages indefinitely. Although bacteriophages are natural products, producing them in high quantities is not natural. Unexpected changes could be introduced. In the case of industrial bacteriophage preparations, permanent monitoring of the production process is seen as mandatory and must be reproducible.

Final bacteriophage preparations must be *pure* (absent of residual contaminating bacteriophages, absent of (other) hosts), *sterile, endotoxin purified* and *pH neutral*. *(Endo) toxin testing* and/or *pyrogenicity testing* of the final products is/are considered necessary. The final *bacteriophage titre* must be tested, as well as the *(storage) stability* (and conditions).

Assessing *pharmaco*-*kinetics* of the bacteriophage preparations (in relation to the application format, under relevant conditions) is also beneficial. Also the *(adverse) immune response* of the human body should be studied. *In vitro* *modeling* is important to understanding the action of the bacteriophages.

When bacteriophages are stored in a “therapeutic phage bank”, it would be interesting to compare the quality management applied in such master bacteriophage banks with that applied in human cell banks.

In contrast to this rationale, counterarguments state that no elaborated bacteriophage quality and safety documentation is necessary since the safety of bacteriophages has been proven through their long-standing historical use. Bacteriophages are the most abundant form of “life” on earth and are even older than bacteria. If bacteriophages were pathogenic to humans, so goes the argument, it would be publicly known by now. According to this way of thinking, efficacy is all that must be tested and human clinical trials should be conducted. Historical data related to bacteriophage therapy were not, in most cases, generated in accordance with western research standards. Most of these data were collected through “open” clinical trials in eastern countries and lack any written decent reports or data audits. Therefore, in order to be useful, these historical data must be validated. In view of this, it has been suggested to (partially) fall back on these historical data for documenting bacteriophage safety. Efficacy must be proven through standardized clinical trials.

In any case, the documentation of therapeutic bacteriophages is something to take seriously. Data obtained through scientifically sound clinical trials must live up to western standards. It is important to explain (especially to regulators) what is known about bacteriophages and their therapeutic use and to define acceptable risks of bacteriophage therapy.

In the future, basic sequencing research should be performed to see whether lytic bacteriophages could ever turn into a lysogenic state. In addition, the question of how bacteriophages can adapt to existing natural beneficial bacteria—and what the consequences of such an adaptation could be—should also be addressed. It remains uncertain whether and how bacteriophages can infect eukaryotic cells. Basic studies in vitro have to be validated in vivo. The performance of bacteriophages in vivo can vary from their in vitro activity. In addition, blood–brain barrier crossings must be studied in humans as well as in mice. Evolutionary models have also proven to be important to the study of specific interactions between bacteria and bacteriophages.

##### Natural Bacteriophages Comparable to Vaccines or Toxins?

From a regulatory point of view, bacteriophages are most similar to a particular type of biological medicinal products, namely *vaccines*. More in particular, bacteriophage cocktails used in humans need to be updated over time, especially when bacterial resistance develops (as is the case with the flu vaccine). For that reason, some bacteriophage companies are now liaising with the vaccine unit of the European Medicines Agency (EMA).

However, bacteriophages are not “regular” *vaccines* that are mostly used preventive to produce active immunity and therapeutic only in particular cases. Bacteriophages on the contrary are antimicrobials, with a secondary competence of boosting the immune system, be it in a non-specific manner. Since bacteriophages (or their lysates) can boost the immune system (in different ways), they can effectively be seen as “therapeutic vaccines”. Using bacteriophages in this way implies the concept of “auto-vaccination” via bacteriophages, meaning vaccinating patients with their own bacteriophages. Parallels exist in this sense between bacteriophage therapy and tumor vaccination. In the latter, the patients’ own tumor tissue is taken for preparation of the vaccine and the patient’s own immune response towards its own tumor is modified. This immune response should be self-limiting since the reaction has to stop when the tumor is gone.

Triggering the immune system is not necessarily positive for the bacteriophage itself since it can be eliminated by the immune system of the patient before destroying the bacteria. On the other hand, bacteriophages can be used to test the state of immunity of the patient in general.

With respect to the category of *toxins*, bacteriophages are not toxic and hence they are not to be considered *toxins* as described in the Directive 2001/83/EC.

#### Views on Bacteriophages and the Definition of an ATMP (Box 3)

ATMPs are defined in Directive 2001/83/EC as complex therapeutic products. Natural bacteriophages can be considered as *complex products*. Once administered to the patient, control over the stability of the bacteriophage products is lost. When bacteriophages are applied in the wound-bed of the patient, bacteriophages can replicate in bacteria and bacteriophage-variations and mutations can develop. Pharmacokinetics of administered bacteriophages (absorption process) is very complex. Sequencing and determining the exact function (e.g., proteomics) is also complex, as is determining the way bacteriophages realize their therapeutic effect.

However, the actual categories within the ATMP framework (*products for gene therapy, somatic cell therapy or tissue engineering*) are not suitable to natural therapeutic bacteriophages. For instance, natural bacteriophages are not gene therapy medicinal products since they are not genetically modified. For obvious reasons, bacteriophages are not considered somatic cells therapy medicinal products nor tissue engineered medicinal products.

For most complex therapeutic products, a precise legal definition is required. Since natural bacteriophages are already present in nature and in our body, it is questionable whether such a definition is necessary for bacteriophages. The complexity is of a technically different nature than gene or somatic cell therapy. Bacteriophages could be compared to more widely used strategies for improving microbial ecology such as probiotics.

Differences and/or Similarities of Bacteriophage Therapy Versus Antibiotic Therapy.

At the product level, antibiotics are (mostly) synthetically prepared chemical products, although antibiotic compounds isolated from nature exist as well. Natural bacteriophages are (by definition) natural “products”.

In terms of function, both antibiotics and bacteriophages modify (indirectly) human physiological functions by destroying pathogenic bacteria. Some antibiotics act at the genetic level while others block specific metabolic pathways. Therapeutically relevant bacteriophages, which are lytic natural bacteriophages, kill bacteria by other mechanisms. Such bacteriophages destroy the bacterium “from the outside” by massively perforating the cell membrane, or “from within” by multiplying within the bacterium and eventually being released from that bacterium. Some modern antibiotics cause lyses of the bacteria as well. Endotoxins are released within the patient through lyses or bacterial cell death in general. In the case of antibiotics, this release almost never causes a major problem for a patient confronted with major (resistant) infections, which is what can be expected for bacteriophage therapy as well.

An important difference between bacteriophages and antibiotics is that bacteriophages have a much more specific, targeted action. The broadest-spectrum bacteriophage will never execute as wide an action as the most targeted antibiotic product does. Therefore, bacteriophages do not disturb the natural flora as much as antibiotics do. However, bacteriophages’ high specificity can also be considered a negative factor for clinical application. Another important difference is that bacteriophages are able to diffuse in small numbers to the site of bacterial infection and then multiply only when needed. Antibiotics, on the other hand, must be administered in high doses right at the site of treatment, which may cause collateral damage to the patient.

In contrast to antibiotics, bacteriophages can cross the blood–brain barrier (in small quantities) and perform their action (massively) once the target bacteria are reached. Another advantage of bacteriophages over antibiotics is the reduced risk for development of resistance. The amount of bacteriophages does not decrease when approaching the bacterial target. Distinct from antibiotics, bacteriophages have an additional capacity to act on biofilms since their lysins can destroy the biofilm.

In view of those differences and similarities, most experts agree that bacteriophages and antibiotics should be used complementarily/synergistically.

#### Views with Respect to (Marketing) Authorization for Bacteriophage Therapy

##### Two Regulatory Pathways

According to the interviewees, two (complementary) regulatory pathways should be defined for bacteriophage therapy.

The first is a regulatory path for a uniform product market placement of natural bacteriophage-based products. Since bacteriophages are regarded as human medicinal products, the actual legal framework for human medicinal products in Europe is applicable, implying submitting a full product dossier (complying with Directive 2001/83/EG) and conducting large-scale expensive clinical trials. This path imposes several hurdles: (1) The high financial threshold cannot easily be overcome by public stakeholders such as hospitals without financial support from other (government) sources. (2) The current Directive 2001/83/EG provides insufficient technical guidance and legal certainty for the development of products for natural bacteriophage therapy. (3) The primary aim of public stakeholders is in fact not a real “market placement” or “marketing authorization” of a bacteriophage-based (or any other) product. (4) One major risk of such market placement of bacteriophage based products is the development of large-scale resistance, at least when use is widespread and uncontrolled. One suggestion could be to update a standard bacteriophage cocktail preparation on a yearly basis once it is on the market and resistance begins to develop, as is done with the flu vaccine.

The second regulatory path should imply an approach applicable to tailored, patient-specific treatments.

The question arises as to whether hospitals applying a tailored bacteriophage therapy approach in close collaboration with microbiological labs should be excluded from the conventional marketing authorization requirement as described in Directive 2001/83/EG and national laws. Certain non-profit-driven hospitals often possess clinical expertise to provide bacteriophage therapy but lack the financial capacity and interest to engage themselves in large-scale market placements of authorized bacteriophage products. In addition, the bacteriophage itself is not a “product to be brought to the market” (citing the wordings of Directive 2001/83/EC) and the tailored hospital-based bacteriophage therapy approach is the only approach that in reality fully exploits the clinical potential of a therapeutic bacteriophage. Bacteriophage therapy is in fact a therapeutic concept.

If patient-specific use of bacteriophage therapy in hospitals is made exempt from the regulatory framework designed to receive marketing authorization (similar to the hospital exemption rule within the regulatory framework of advanced cell and tissues, ATMPs), quality and patient safety must be guaranteed. It is also argued that not only hospitals but also industry, with specific approval from regulators, should in theory be able to deliver “out-of-frame” and “tailored” bacteriophage preparations to patients and hospitals on a per-request basis. However, the difficulty and expense of applying the “one product for one patient” model is not cost-efficient.

In view of this, it may be more prudent to regulate bacteriophage therapy via a simplified marketing authorization framework, feasible for hospitals as well, by strictly defining the (often rare) indications for bacteriophages uses. For industries interesting in market approval for bacteriophage cocktails, endeavors to work under the orphan drug legal frameworks should be explored.

##### Over-the-Counter Distribution

In view of distribution, there are arguments for a very “liberal” distribution model for natural bacteriophages intended for therapeutic use. Some argue that “over-the-counter” distribution of bacteriophages will increase resistance development, while others argue that "over-the-counter" distribution should be possible on the grounds that solely hospital-based use cannot preclude the development of resistance. Next, others claim that limiting distribution to those who are tested is unrealistic, since testing all patients before allowing them to take bacteriophages would be expensive and economically infeasible. Such pre-testing is not readily available for other conventional drug therapies either.

A consensus solution could be to organize over-the-counter distribution of standard bacteriophage-based cocktail products specifically selected for non-life-threatening infections while leaving treatment in life-threatening situations to tailored bacteriophage-based products in a hospital environment. Most ideal would be hospital-based (lab-linked) and accessible (cheap) use of natural bacteriophage-based products. National “bacteriophage therapy centers” (scientific boards included) as are now being set up in Brussels, could be of great value, in preference when linked to a “therapeutic bacteriophage bank” (e.g., DSMZ—http://www.dsmz.de) where specific bacteriophages could be stored and produced as needed. For any treatment, patients must be tested for the best strain match. Individualized approaches and flexibility for physicians to treat patients via personalized schemes should be central.

#### Views on an Adapted or New Legal Framework for Bacteriophage Therapy

Stakeholders are convinced of a need for a dedicated (new) regulatory framework for bacteriophage therapy that acknowledges the specific properties of bacteriophages and their bacterial interaction as well as the role of hospitals as providers of bacteriophage therapy. As explained above, bacteriophages are uniquely different from conventional human medicinal products (such as chemical substances, somatic cell therapy products and gene therapy medicinal products, among others) currently regulated under existing frameworks. In view of the fact that even products for homeopathy have a dedicated legal framework, some question why bacteriophage therapy is not regulated in a specified, dedicated way.

An adapted regulatory framework could, for instance, be inspired by the existing legislation governing “advanced cellular and genetic therapy” (ATMPs), where regulators took a binary approach towards industrial as well as hospital-based use of cell and gene products and therapies.

Next to the two-way regulatory paths, a new or adapted framework for bacteriophage therapy must in addition take into account the different trajectories for storing and making available therapeutic bacteriophages. (1) A first possibility would be to use the patient’s own bacteriophages in a tailored approach for that individual patient. No long-term storage of bacteriophages would be necessary, but on-site testing facilities would have to be present wherever this kind of tailored therapy is offered (“bacteriophage therapy centers”). (2) A second approach comprises the isolation and storage of well-defined (GMP-produced) therapeutic bacteriophages in a bacteriophage bank, which would then be distributed as needed. Such bacteriophages can represent starting materials for the preparation of a cocktail that could be used for combating broad-scale bacterial infections, e.g., in refugee camps confronted with dysentery. At best, different bacteriophages targeting the same bacterium would be collected and, if necessary, provided for therapeutic use, minimizing resistance issues. (3) Such therapeutic bacteriophages stored in a bank could be ordered as well by a physician for tailored-use within a hospital.

An adapted or new regulatory framework for bacteriophage therapy must guarantee safety and quality. Regulatory conditions that govern the production of human medicinal products (e.g., Good Manufacturing Practices) impose high costs and are perhaps not necessary to increase the safety of bacteriophage-based products. Instead, specific guidelines solely directed at quality and safety of bacteriophage preparations should be developed, harmonized and controlled.

If regulators and legislators are to adapt existing legislation (and its interpretation), public as well as private stakeholders must agree on what type of pathways and approaches need to be developed. All partners in these discussions will eventually come to a consensus understanding on the use of therapeutic bacteriophages and that this understanding will serve as a basis for moving forward in a constructive way.

While regulatory frameworks are (and should be) the product of negotiations with regulators and legislators, the negotiation process takes time; time that is precious given the acuteness of the problems faced. In view of the fact that EMA recognized the regulatory framework of biological medicinal products as applicable to bacteriophages, this regulatory pathway might just be the best place to start for further elaboration. Since the regulatory frameworks relevant to the development of bacteriophage therapy are actually more reasonable in, e.g., Australia, Canada, it is time for Europe and individual European countries to take action. At the same time, an international platform should ensure that international harmonization develops.

### Patenting Bacteriophage-Related Applications

Isolated, therapeutic bacteriophages can in theory be patented when a complete, well-defined documentation package is available for the specific bacteriophage(s). This package comprises data related to the genome sequence, pre-clinical information, specific functionality, and specific application, among other features. Inventive steps can be defined on the basis of molecular characteristics, application methodology and eventually production procedures. In practice, patents on bacteriophage products are important tools for attracting investors to new companies keen on developing therapeutic bacteriophages.

Similar to most vaccine patents, a patent for a regularly updated bacteriophage cocktail can also be sought.

Companies interested in placing therapeutic bacteriophages on the market take care of IP: they first choose their most appropriate market niche, gain experience from a regulatory point of view and acquire a first return on investment. In a next step, after building more experience on the subject, expansion to other markets can proceed. IP protection is important in order to be able to develop this pathway.

## Discussion

Directive 2001/83/EC defines a human medicinal product, the types of action, its sources and its starting materials. This definition is formulated rather broadly, encompassing natural bacteriophages. For instance, for the products covered by its scope, the Directive does not differentiate between “*direct*” or “*indirect*” therapeutic actions. Bacteriophages generate their action on the patient in an indirect way, similar to antibiotics. Bacteriophages destroy the bacterial pathogen and consequently eradicate or decrease the pathogenic bacterial load in the patient (Payne and Jansen 2003).

It is clear from our analysis that natural bacteriophages fit into the definition of a “biological medicinal product” (Box 2). However, different *biological sources* for the production of a therapeutically active bacteriophage are possible. Since the definition of a biological medicinal product does not limit the types of potential biological sources, therapeutic bacteriophages also comply with the definition of a biological medicinal product. However, therapeutic bacteriophages do not fit into the Special Frames (indicated in the Directive 2001/83/EG) applicable to biological medicinal products, such as vaccines, toxins and serum-derived products.

In view of the applicability of the ATMP definition (in Regulation 1394/2007) to bacteriophages, it is not clear whether bacteriophages are “complex therapeutic products with technical specificities requiring precise legal definitions” (as is true for ATMPs). In a sense, the regulatory pathways developed for “natural” ATMPs might provide a historical reference point. Products used in somatic cell therapy, when substantially manipulated or used in a non-homologous way, are classified as ATMPs. The human medicinal product Directive 2001/83/EC defines “cultivation”, for instance, as a substantial manipulation. Consequently, natural bacteriophages, when cultivated, could also be seen as fitting within the ATMP framework since they would, according to this definition, be substantially manipulated.

## Impact of Classifying Natural Bacteriophages as Human Medicinal Products

The development of a human medicinal product, either as a biological medicinal product (Dir 2001/83/EG) or as an ATMP (Dir 2001/83/EG and Regulation 1394/2007) requires huge investments of time and money. The non-profit sector and the diverse interested small and medium enterprises can hardly afford this pathway without external investments. Therefore, there is a need for products like natural bacteriophages to be exempted from the scope of the regulatory framework applicable to human medicinal products, more specific Directive 2001/83/EG and Regulation 1394/2007, depending on whether bacteriophages are seen as biologics or ATMPs.

One way would be not to formulate the therapeutic action of the bacteriophage as a primary mode of action, arguing that such a product is not a human medicinal product. However, this is not the most optimal scenario when the ultimate goal of the exercise is “to bring therapeutic bacteriophages to the patient” (Międzybrodzki et al. 2012; Soothill 2013; Wittebole et al. 2013). In addition, by reviewing the definitions, all reviewers acknowledged that a bacteriophage may fit into the definition of a human medicinal product.

Another way is to use exemptions within the existing regulatory framework for human medicinal products. If natural bacteriophages are considered ATMPs, the ATMP Regulation 1394/2007 is applicable. This framework only specifies certain categories, human somatic cell therapy, gene therapy and tissue engineering. A specific category “viral therapy” could theoretically be introduced under this ATMP framework. In any case, the ATMP Regulation 1394/2007 provides a possibility for hospitals to be exempted from a stringent centralized marketing authorization, referred to as the “hospital exemption” (Art. 28 of Regulation (EC) No. 1394/2007). National rules apply to hospital exempted-ATMPs. However, if present, such rules are in any case specifically designed for cell and tissue based therapies, not bacteriophage therapy. In addition, often such national rules require similar GMP as requested for fully centralized marketing authorization dossiers. Therefore, a specific Directive covering natural bacteriophage therapy (to be implemented in national laws specific for bacteriophage therapy) is desirable.

If natural bacteriophages are considered biological human medicinal products, Directive 2001/83/EG applies. Unfortunately, the Directive 2001/83/EG (currently) does not provide for a hospital exemption (as in the ATMP Regulation). But Article 3 (Paragraph 1) of Title II of the Directive states that it “*shall not* apply to any medicinal product prepared in a pharmacy in accordance with a medical prescription for an individual patient (commonly known as the magisterial formula).” However, a (hospital) pharmacist is not supposed to use non- (EU) licensed products as components for magisterial preparations. Since natural bacteriophages are not licensed products at the moment, this potential pathway could be difficult to implement.

Another way to escape the marketing authorization requirement is to consider the scope of that Directive. Article 2, Par. 1 (Title II) of the Human Medicinal Product Directive states that it “shall apply to medicinal products for human use *intended to be placed on the market* in Member States and either *prepared industrially* or manufactured by a method *involving an industrial process*”. As highlighted by the interviewees, a tailored (natural) bacteriophage production (Merabishvili et al. 2009), performed within a hospital for use on particularly defined patients can hardly be seen as an “industrial production”. In addition, the therapeutic, in-house use of these produced bacteriophages is not “market placement”. Hospitals are not interested in producing human medicinal products for the purpose of obtaining a “marketing authorization” for further distribution. For these reasons, and analogous to the logic developed in the field of the cellular ATMPs, the tailored production and therapeutic use of natural bacteriophages on humans would appear *not* to be covered by the scope of the Human Medicinal Product Directive. The industrial productions of uniform bacteriophage products intended for European market placement, on the other hand, *are* covered by the scope of this Directive.

If natural bacteriophages would be covered by the scope of the Directive 2001/83/EG, a new hospital exemption needs to be designed for biological human medicinal products, accompanied by a specific Directive for bacteriophage therapy (to be implemented in national laws).

It is clear from the above that there is a regulatory gap for natural bacteriophage therapy. While regulators are responsible for applying a regulation, the regulation itself can only be changed through legislative action, which is in this case highly needed to guarantee a timely, flexible and sustainable way of introducing bacteriophage therapy in Europe.

**Fig. 2:**
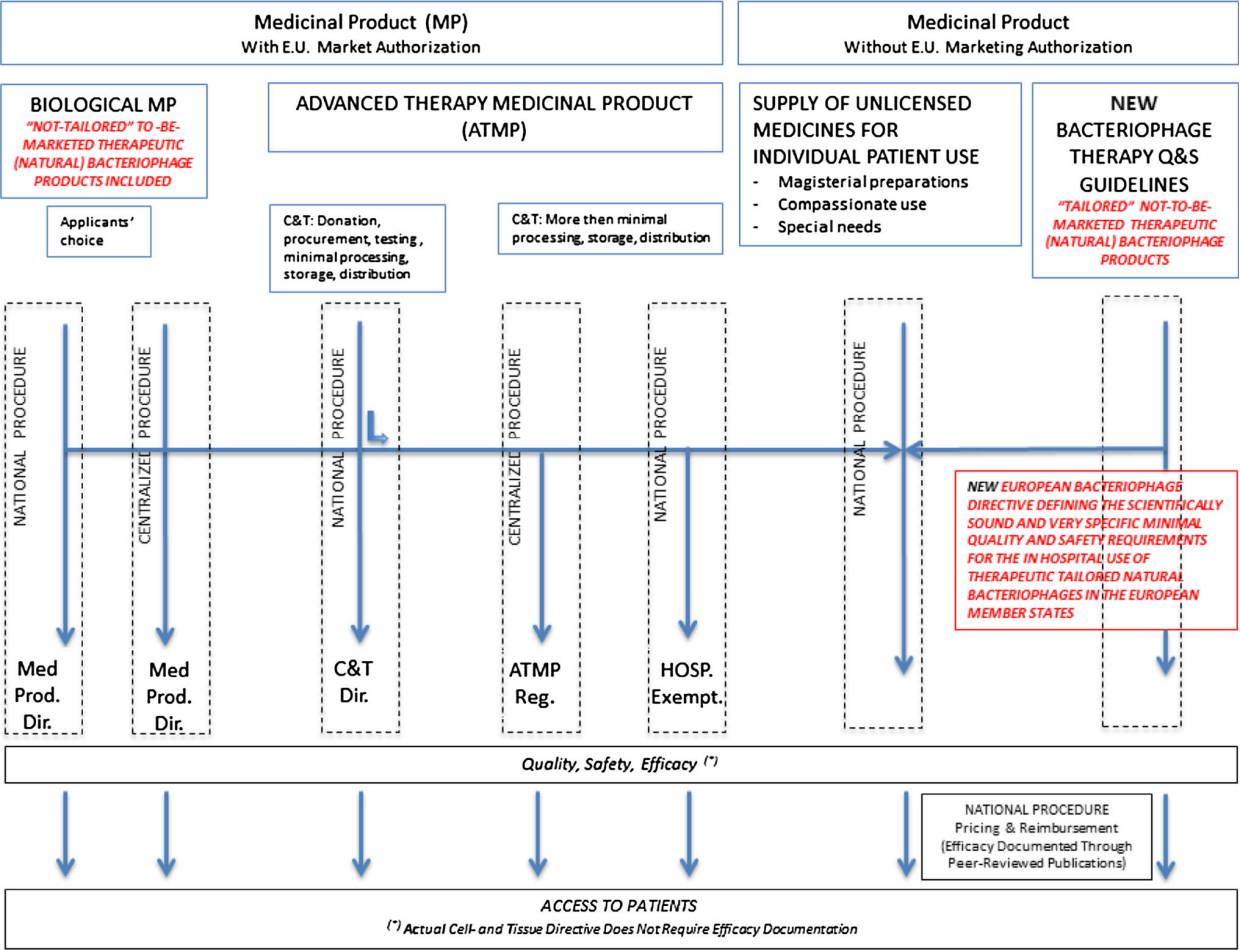
Proposal for a new European directive for bacteriophage therapy

The appropriate legal action we suggest is a European wide Directive for Natural Bacteriophage Therapy. Such Directive should regulate documentation requirements of safety, potency, purity and toxicity, specific in the context of hospital based patient-tailored (natural) bacteriophage therapy. (Industrial) stakeholders aiming to bring pharmaceutical products based on natural bacteriophages to the market could be exempted from the scope of the new Directive and follow the classical medicinal product approach instead (see Fig. 2).

The creation of a bacteriophage-specific Directive could find inspiration on the evolution of what has transpired the last several decennia in the field of human cell and tissue engineered products. As early as the seventies, hospitals were using processed human cells and tissue in treatments for their patients, in accordance with the respective national legislation, until the European Human Cell and Tissue Directive was published in 2004 (Directive 2004/23/EC of the European Parliament and the Council of 31 March 2004). This Directive still applies to all work with human body material today and focuses mainly on the hospital-based development and use of cellular products. A few years later, in 2007, the ATMP Regulation (EC) No. 1394/2007 came into force. This ATMP regulation focused (and still does) mainly on industrial work with human bodily material. At the same time, it allows for a “hospital exemption”. The hospital exemption applies to non-industrial, tailored and hospital-based clinical use of cell and gene based ATMPs. Industrial ATMPs meant for market placement are regulated at the European level while hospital-based (non-industrial) productions are regulated at the national level.

In a similar way, the bacteriophage-specific regulatory framework with its specific Directive should (1) distinguish between hospital-based (tailor-made) use of natural therapeutic bacteriophages in patients on the one hand and industrial production and distribution of uniform bacteriophage products on the other, (2) define specific quality and safety criteria relevant to the use of natural bacteriophages on patients (Merabishvili et al. 2009; Wright et al. 2009), (3) define a specific efficacy documentation package relevant to (natural) bacteriophages, (4) make it possible to give patients in need instant access to natural bacteriophage therapy (Caplin 2009), (5) only define requirements relevant to natural bacteriophages, and (6) fully exploit the co-evolutionary aspects of natural bacteriophages (Krylov 2011; Levin and Bull 2004; Scanlan and Buckling 2012).

Close dialogue, open discussions and information exchange with the EMA and national authorities is crucial. It is thus of high importance that regulators and legislators (Members of Parliament) be persuaded of the prudence of a dedicated Bacteriophage Therapy legal framework for Europe.

## Conclusions

A dedicated European Bacteriophage Therapy Legal Framework is a prerequisite for paving the way to the smooth introduction of natural bacteriophage therapy into western medicine. If Europe refuses to support the short-term (safe) implementation of “hands-on” bacteriophage therapy in its member states, the national authorities of the member states should step into assert their responsibility in this respect. Antibiotic resistance is an acute problem, both in public health terms and socio-ethical terms. 25 000 Europeans die each year as a direct consequence of untreatable bacterial infections (Ackermann 2012). There is an urgent need for national bacteriophage therapy centers. Industry can play an important role in this. When bacteriophage therapy centers are unable to (financially) launch themselves, national governments should provide sufficient support and/or stimulate the creation of new pharmaco-economic environments.

**Box 1.** Definition of a Human Medicinal Product within the Directive 2001/83/EC of the European Parliament and the Council of 6 November 2001 on the Community code relating to medicinal products for humans use. Consolidated | 2001L0083-EN-21.07.2011-010.002-1. Words written in *Italic* are subject to interpretation, as discussed in the manuscript. According Art. 1(2) of the Medicinal Product Directive 2001/83/EC, a Human Medicinal Product is a *substance* or a *combination of substances* presented as having properties for *treating* or *preventing* disease in human beings. According the same Directive, a Medicinal Product *can also be* a *substance* or a *combination of substances* which may be used in or *administered* to human beings either with a view to *restoring*, *correcting* or *modifying* physiological functions by exerting a *pharmacological*, *immunological* or *metabolic* action, or to making a *medical diagnosis*. The *substance* referred to in the Directive is *any matter* irrespective of origin which may be: human, e.g., human blood and human blood products; animal, e.g., *microorganisms*, whole animals, parts of organs, animal secretions, toxins, extracts, blood products; vegetable, e.g., microorganisms, plants, parts of plants, vegetable secretions, extracts; chemical, e.g., elements, naturally occurring chemical materials and chemical products obtained by chemical change or synthesis.

**Box 2.** Definition of a Biological Medicinal Product. Words written in *Italic* are subject to interpretation, as discussed in the manuscript According to Part I Module 3 (3.2.1.1) of the Medicinal Product Directive 2001/83/EC, a Biological Medicinal Product is a Medicinal Product of which the active substance is a *biological substance*. A biological substance is a substance that is *produced by* or *extracted from* a *biological source* and that requires for its *characterization* and the *quality determination* a combination of physico-chemical-biological testing, together with the production process and its control. The Directive provides specific requirements for particular biological medicinal products such as *vaccines*, *toxins* and *sera*, in particular, for agents used to produce active immunity, such as cholera vaccine, BCG, polio vaccines, smallpox vaccine; agents used to diagnose the state of immunity, including in particular tuberculin and tuberculin PPD, toxins for the Schick and Dick Tests, brucellin; and agents used to produce passive immunity, such as diphtheria antitoxin, anti-smallpox globulin, antilymphocytic globulin. According the Directive (Part I Module 3 (3.2.1.1), the *starting materials* of a Biological Medicinal Product shall mean *any* substance of biological origin such as microorganisms, organs and tissues of either plant or animal origin, cells or fluids (including blood or plasma) of human or animal origin, and biotechnological cell constructs (cell substrates, whether they are recombinant or not, including primary cells).

**Box 3.** Definition of Advanced Therapy Medicinal Product (ATMP) from Regulation (EC) No 1394/2007 of the European Parliament and the Council of 13 November 2007 on advanced therapy medicinal products and amending Directive 2001/83/EC and Regulation (EC) No 726/2004 | Official Journal of the European Union | 10.12.2007 | L324:121-137. Words written in *Italic* are subject to interpretation, as discussed in the manuscript. The Regulation 1394/2007 on ATMPs (Recital 3) describes ATMPs as *complex therapeutic products* with *technical specificities* requiring *precise legal definitions*. Under the ATMP Regulation, Products for Somatic Cell Therapy, Tissue Engineered Products as well as Gene Therapy Medicinal Products (GTMPs) are classified as ATMPs. According to Part IV of Annex I to Directive 2001/83/EC, a Gene Therapy Medicinal Product is a biological medicinal product that has the following characteristics: (a) It contains an active substance which contains or consists of a recombinant nucleic acid used in or administered to human beings with a view to regulating, repairing, replacing, adding or deleting a genetic sequence; (b) Its therapeutic, prophylactic or diagnostic effect relates directly to the recombinant nucleic acid sequence it contains, or to the product of genetic expression of this sequence. Gene therapy medicinal products shall not include vaccines against infectious diseases.
